# Web-based formative assessment through clinical cases: role in pathophysiology teaching

**DOI:** 10.1186/s12909-021-02691-y

**Published:** 2021-04-30

**Authors:** Nerea Fernández Ros, Felipe Lucena, Mercedes Iñarrairaegui, Manuel F. Landecho, Patricia Sunsundegui, Carlota Jordán-Iborra, Iñigo Pineda, Jorge Quiroga, Jose Ignacio Herrero

**Affiliations:** 1 Department of Internal Medicine, Clínica Universidad de Navarra, Pamplona, Spain; 2 Instituto de Investigación Sanitaria de Navarra (IdiSNA), Pamplona, Spain; 3 Liver Unit (Department of Internal Medicine), Clínica Universidad de Navarra, Av Pío XII, 36, 31008 Pamplona, Navarra Spain; 4 Centro de Investigación Biomédica en Red de enfermedades hepaticas y digestivas (CIBERehd), Madrid, Spain

**Keywords:** Web-based, Formative assessment, Pathophysiology, Case-based learning

## Abstract

**Background:**

Active learning strategies such as formative assessment through clinical cases may help to get a deeper learning. We have studied the effect of this kind of online formative assessment in pathophysiology teaching.

**Methods:**

Seven brief clinical cases were used to give formative assessment in the first semester of a pathophysiology course. To evaluate its effect on learning, we analyzed the proportion of students that passed the end of semester exam with a score above 60 over 100. We also analyzed the effect of the intervention according to the students’ previous academic performance.

**Results:**

Ninety-six students participated in the study and sat the exam. Sixty-five of them passed it. Students that passed the exam had a higher previous academic performance and had done a higher number of exercises of formative assessment, both in univariate and multivariate analysis. The participants were divided in three groups, according to their previous academic performance. In the intermediate group, the number of cases done by the students who passed the exam was significantly higher than in those who did not pass it (median: 4 versus 0; *P* = 0.009).

**Conclusion:**

Formative assessment through web-based clinical cases was followed by an improvement of the academic results in pathophysiology, mainly in students with intermediate performance.

## Background

One of the challenges of teaching pathophysiology is to obtain a deep learning of the subject. Active learning strategies help to obtain such a deep learning, and reinforce knowledge, as they promote the process of high order thinking. These active strategies may include teaching through directed cases [1, 2], supplementary cases [3, 4] or through the assessment based on clinical cases [5–8].

Assessment is not only a tool to measure students’ performance, but it is also useful for enhancing their learning. Thus, assessment has also a formative role. Formative assessment has been described as a process of appraising, judging or evaluating students’ performance and use it to improve their competence [9]. Formative assessment reinforce the central and active role of students in their own learning through a constructive feedback that precedes the summative assessment [10].

The use of computer-based online formative assessment can be self-administered, and is beneficial for the learning process, because it provides immediate feedback [11]. Online formative assessment improves learning in different subjects of undergraduate medical teaching such as Radiology [7], Anesthesiology [5], Endocrinology [8], or musculoskeletal system [11]. On the other hand, Cassady and Gridley did not find a consistent effect of formative assessment on summative test when they used past performance as a covariate [11].

Thus, the aim of this paper was to report the effect of computer-based formative assessment through self-administered questions about clinical cases in the teaching of Pathophysiology to third-year medical students.

### Conceptual framework

Teaching Pathophysiology in a large group (200 learners) at our university is based on didactic lectures, including flipped learning recently [12]. Formative assessment has been shown to improve students’ performance in undergraduate medical education [5, 7, 11]. Thus, we decided to introduce formative assessment through brief clinical cases and to test its effects. The results of this method of active learning may depend on individual characteristics. Our main objective was to improve the learning experience of all students, mainly of those with mid and poor performance. Thus, we examined the effect according to their gender, previous academic results and academic delay.

## Methods

### Participants and settings

The curriculum in the School of Medicine of the Universidad de Navarra (Pamplona, Spain) consists of three preclinical and three clinical years. Participants were third-year medical students from the Universidad de Navarra that were enrolled in the Pathophysiology course in 2019–20. Pathophysiology is a whole-year course divided in 4-month semesters. The first semester runs between September and December and includes cardiovascular, renal and blood pathophysiology; the second semester (January to April) includes respiratory, neurological, endocrine, metabolic and liver and gastrointestinal pathophysiology. The subject is taught by members of the Department of Internal Medicine. At the end of the first semester, the students complete an exam. Those who obtain a score above 60 out of 100 do not need to be examined for this first semester topics in the final exam. The exam includes a test with 100 multiple-choice questions (four choices for every question). The final score is obtained after substracting one third of the number of wrong answers to the number of correct answers.

### Intervention

In the first semester of 2019–20, an online-based formative assessment was used. Clinical cases were provided to the students through a Google Forms questionnaire. They were available few days after the explanation of the topic, without restriction of time and number of attempts. After the description of a clinical scenario, the students had to answer multiple-choice questions to obtain more information about the case, until reaching the diagnosis. Every case included 3–4 questions that could be responded in approximately 5 min and, after responding to each of them, an immediate feedback was offered to the student, but not to the professor. This feedback included a pathophysiological explanation about the right and wrong options. Seven clinical scenarios were provided: acute pericarditis with cardiac tamponade, acute myocardial infarction, nephritic syndrome, chronic renal disease with acute kidney injury, megaloblastic anemia, autoimmune thrombocytopenia and hypersplenism.

We studied whether the number of cases done by the students was associated with their performance at the end of semester exam. Gender, the existence of academic delay and past academic performance were studied as potential covariates. Academic delay was defined as an age above 20 years at the beginning of the academic year (born in 1998 or earlier). Past academic performance was analyzed through the performance in the end of semester exam in General Pathology (GP). This subject is taught in the second year (January to April) by staff members of the Department of Internal Medicine. This 4-month course is an introduction to Pathophysiology and is mostly devoted to the explanation of etiology and pathogenesis of disease.

### Ethics statement approval and consent to participate

The study was conducted in accordance with the Declaration of Helsinki. It was approved by the School of Medicine of the Universidad de Navarra and by the Ethics Committee for Research of the Universidad de Navarra (project 2019–114). Students gave their informed consent for their participation in the study and were not compensated for it. Data from the students were recorded in a coded database, without personal information.

### Statistical analysis

Continuous variables are expressed as median (quartile range) and categorical variables as number (percentage). The dependent variable was whether the students obtained a score above 60 over 100 (the minimum score to pass the exam) in the end of first semester exam. The independent variables were gender, academic delay, score in the exam of GP (in May 2019) and the number of cases done. Groups were compared with the Mann-Whitney test (categorical variables) and the Chi-square test (categorical variables). A multivariate logistic regression analysis was done with the variables showing a *p* value < 0.2 in univariate analysis.

A similar analysis was performed after dividing the class in three groups according to their previous scores in GP.

All data were analyzed with the software IBM SPSS Statistics for Windows version 20.0 (IBM Corp., Armonk, NY). A *p* value below 0.05 was considered statistically significant.

## Results

In 2019–20, 194 students were enrolled in the Pathophysiology course and 98 (50.5%) of them gave their consent to particpate in the study. Seventy of them (71.4%) were females and 28 (28.6%) were males. Only 11 participants (11.2%) had an academic delay. Their median score in GP had been 64.9 (56.8–74.5) out of 100. There were no significant differences in GP scores between males and females and between students with or without academic delay (data not shown). The median number of cases done by each participant was 3.5 (0–7).

### Analysis of factors associated to passing the exam

Ninety-six participants sat the end of semester exam of Pathophysiology and 65 (67.7%) of them passed it with a score above 60. In univariate analysis, the score obtained in GP in the previous year and the number of cases of formative assessment done by the students were significantly higher in the students who passed the exam (Table 1).

**Table 1:** Factors associated to passing or failing the end of semester exam of pathophysiology

	Passed (*N* = 65)	Failed (*N* = 31)	*P* value
Gender			0.229
Male	16 (57.1%)	12 (42.9%)	
Female	49 (72.1%)	19 (27.9%)	
Academic delay			0.326
Yes	6 (54.5%)	5 (45.5%)	
No	59 (69.4%)	26 (30.6%)	
Score in General Pathology	70 (63–78)	57 (45–63)	< 0.001
Number of cases done	5 (1–7)	0 (0–2)	< 0.001

In multivariate analysis score in GP in the previous year (odds ratio for each 10 points: 2.891; 95% confidence interval: 1.692–4.940; *P* <  0.001) and the number of cases done (odds ratio: 1.393; 95% confidence interval: 1.101–1.763; *P* = 0.006) were independently associated to the endpoint of passing the exam.

### Analysis of academic results according to previous academic performance

We divided the participant students in three groups, according to their scores in the end of course exam in GP in the previous year. Group 1: scores up to 60 over 100 (*N* = 29), group 2: scores between 60 and 71 (*N* = 31) and group 3: scores higher than 71 (*N* = 32). Within each of these groups, we studied if the endpoint of obtaining a score above 60 in the exam of pathophysiology was related to a higher score in GP and / or to having done a higher number of formative assessment cases. In group 1, there were not differences either in the score obtained in GP or in the number of cases done between those who achieved the endpoint or not. In group 2, the students who achieved the endpoint had done more cases than those who did not, but their scores in GP did not differ. In the group 3, there were also no differences in the GP score or in the number of cases done between those who achieved the endpoint and those who did not, but only one of them failed the exam. Thus, we also analyzed if there was a difference between those participants in the group 3 who achieved a score in the highest quartile of the class (above 77 over 100) or not. Students in the highest quartile had better scores in the GP exam in the previous year, but the number of cases they did was similar to those who did not obtain such a score (Table 2).

**Table 2:** Scores obtained in General Pathology and number of formative assessment cases done by each student. Comparison between those who obtained a score above 60^a^ in Pathophysiology and those who did not

Group 1 (General Pathology score below 60)			
	Pathophysiology score < 60	Pathophysiology score ≥ 60	*p* value
General Pathology score	50 (41–57)	51 (49–57)	0.512
Number of cases done	0 (0–2)	1 (0–4.5)	0.377
Group 2 (General Pathology score between 60 and 71)			
	Pathophysiology score < 60	Pathophysiology score ≥ 60	*p* value
General Pathology score	64 (63–66)	65 (63–68)	0.428
Number of cases done	0 (0–1)	4 (0.75–7)	0.009
Group 3 (General Pathology score above 71)			
	Pathophysiology score < 77	Pathophysiology score ≥ 77	*p* value
General Pathology score	75 (73–78)	79 (75–85)	0.011
Number of cases done	7 (4–7)	5 (2.75–7)	0.305

^a^71 for the students in Group 3

## Discussion

The results of this study corroborate other authors’ results about the positive effect of the formative assessment through clinical cases on medical students’ learning [5, 6, 8, 11]. This finding may be the consequence of a more active study associated to problem solving, that allows to obtain a deeper learning [13]. Anyway, this effect has not been universally found, as a previous study did not find such a beneficial effect when past performance was used as a covariate [11]. In our study, the effect of the use of the formative assessment tool was independent of previous performance in GP.

On one hand, according to the classification of virtual patients made by Kononowicz et al. [14], case presentation, as used in this study, is adequate to obtain a deeper knowledge, as required in Pathophysiology teaching. On the other hand, the use of virtual patient simulations helps to improve medical skills, but does not seem to improve knowledge, as compared with traditional education [15]. One of the most important issues in this type of pedagogical instrument is the use of feedback since it has an important effect on learning [16]. In our study, the formative assessment tool included an immediate explanation for each question that could help the students to reinforce their knowledge in an easy way. The online resource allowed the professors to give immediate feedback to the students and facilitated the students the freedom to access to it via their own personal devices and home computers. Other design of the resource that allowed obtaining student to student feedback would be probably preferable [16, 17], but also more time-consuming. A recent study has shown that the use of formative assessment case-based through multiple choice questions was well received by students and was followed by an improvement in the academic results in pathpphysiology education [18].

In addition, regarding the amplification of the usefulness of this didactic tool, the analysis of the results of the use of the system and the answers given by the students, may help to predict underachieving students. These results may be helpful for planning interventions to correct the situation [19, 20].

The study shows another interesting finding: the benefit of this resource is centered in students with a median performance. It suggests that students with a low previous performance may probably lack previous knowledge that could be important for their progressive learning. Furthermore, they accessed less frequently to this resource. On the opposite, students with higher scores in GP seem to obtain no benefit of this formative assessment. Most students in group 3 did more cases than the students in the other two groups, making very difficult to find if doing the clinical cases is followed by an improvement in the scores. It is also possible that these students reach a deep knowledge without the help of this formative assessment. However, it is also possible that increasing the complexity of the proposed clinical cases, students with high performance might obtain benefit, too. This is a way of potential improvement of this tool. Our results are comparable with a previous study from our group, where we found that a more active teaching through the flipped classroom environment was followed by a significant improvement in the academic performance that was focused on those students with previous academic performance that was below the median, but without an academic delay [12].

### Limitations of the study

Our study included a limited number of students, and this may limit the generalizability of the results. This is important because the effect of this resource depends on several factors [16], including the subject that is being taught [14] and the design of the cases, questions and feedback. The absence of a control group is another limitation of the study.

## Conclusions

The use of a web-based formative assessment through clinical cases was followed by an improvement in knowledge acquisition assessed using exam scores. Students with a previous performance in the intermediate range seem to benefit more from this learning tool.

## Data Availability

The dataset used in the current study are available from the corresponding author on reasonable request.

## Abbreviation

GP: General Pathology
